# Temporal and Anatomical Host Resistance to Chronic *Salmonella* Infection Is Quantitatively Dictated by Nramp1 and Influenced by Host Genetic Background

**DOI:** 10.1371/journal.pone.0111763

**Published:** 2014-10-28

**Authors:** Wendy P. Loomis, Matthew L. Johnson, Alicia Brasfield, Marie-Pierre Blanc, Jaehun Yi, Samuel I. Miller, Brad T. Cookson, Adeline M. Hajjar

**Affiliations:** 1 Department of Laboratory Medicine, University of Washington, Seattle, Washington, United States of America; 2 Department of Comparative Medicine, University of Washington, Seattle, Washington, United States of America; 3 Department of Microbiology, University of Washington, Seattle, Washington, United States of America; 4 Departments of Medicine and Genome Sciences, University of Washington, Seattle, Washington, United States of America; The Ohio State University, United States of America

## Abstract

The lysosomal membrane transporter, Nramp1, plays a key role in innate immunity and resistance to infection with intracellular pathogens such as non-typhoidal *Salmonella* (NTS). NTS-susceptible C57BL/6 (B6) mice, which express the mutant *Nramp1^D169^* allele, are unable to control acute infection with *Salmonella enterica* serovar Typhimurium following intraperitoneal or oral inoculation. Introducing functional *Nramp1^G169^* into the B6 host background, either by constructing a congenic strain carrying *Nramp1^G169^* from resistant A/J mice (Nramp-Cg) or overexpressing *Nramp1^G169^* from a transgene (Nramp-Tg), conferred equivalent protection against acute *Salmonella* infection. In contrast, the contributions of Nramp1 for controlling chronic infection are more complex, involving temporal and anatomical differences in Nramp1-dependent host responses. Nramp-Cg, Nramp-Tg and NTS-resistant 129×1/SvJ mice survived oral *Salmonella* infection equally well for the first 2–3 weeks, providing evidence that Nramp1 contributes to the initial control of NTS bacteremia preceding establishment of chronic *Salmonella* infection. By day 30, increased host Nramp1 expression (Tg>Cg) provided greater protection as indicated by decreased splenic bacterial colonization (Tg<Cg). However, despite controlling bacterial growth within MLN as effectively as 129×1/SvJ mice, Nramp-Cg and Nramp-Tg mice eventually succumbed to infection. These data indicate: 1) discrete, anatomically localized host resistance is conferred by Nramp1 expression in NTS-susceptible mice, 2) restriction of systemic bacterial growth in the spleens of NTS-susceptible mice is enhanced by Nramp1 expression and dose-dependent, and 3) host genes other than Nramp1 also contribute to the ability of NTS-resistant 129×1/SvJ mice to control bacterial replication during chronic infection.

## Introduction

*Salmonella enterica* serovars Typhi and Paratyphi are human pathogens that cause systemic typhoid fever in infected individuals. Infection with several other serovars, including Typhimurium, Enteritidis, and Dublin, usually results in gastroenteritis in humans (hence the designation non-typhoidal *Salmonellae-*NTS), although a variety of immunological lesions, such as HIV-induced CD4^+^ T cell depletion, genetic defects in oxidative stress pathways (Chronic Granulomatous Disease), and administration of IFN or TNF inhibitors, have been associated with more severe illness in humans [1]. In mice, one inherited form of susceptibility to *Salmonella* infection is associated with a missense mutation in macrophage-encoded solute carrier family 11a member 1 (*Slc11a1*, hereafter referred to as natural resistance-associated macrophage protein, or *Nramp1*), which transports divalent cations out of bacteria-containing phagosomes, potentially inhibiting bacterial metallo-enzymes required for survival of many intracellular pathogens [2], [3]. NTS infection of susceptible (*Nramp1^G169D^*) mouse strains, such as C57BL/6 and BALB/c, leads to acute, systemic infection while resistant mice encoding the wild type Nramp1 allele (*Nramp1^G169^*), including strains 129×1/SvJ, A/J, and C3H, develop long-term chronic colonization [4]–[6]. In both models, *Salmonella* cross the epithelial barrier and colonize the mesenteric lymph nodes (MLN) prior to systemic spread to the spleen and liver [4], [7]–[9]. Susceptible C57BL/6 mice are unable to control systemic replication and succumb to acute infection within a week of infection whereas resistant 129×1/SvJ mice, despite early dissemination of the bacteria, control systemic replication and survive infection [4]. While *Salmonella* is progressively cleared from systemic tissues in 129×1/SvJ mice, MLN colonization persists and can act as a reservoir for relapsing infections [4], [10]. We hypothesized that C57BL/6 mice genetically engineered to express the resistant *Nramp1^G169^* allele would survive the acute phase of infection and establish persistent *Salmonella* colonization, allowing us to study protective immunological responses in chronically infected mice. Interestingly, we found that expression of *Nramp1^G169^* was sufficient to control bacterial replication in MLN but not in systemic tissues even in transgenic C57BL/6 mice overexpressing *Nramp1^G169^*, which has been shown to promote survival during acute *Salmonella* infection [11], [12]. Therefore, these data also reveal that resistant mouse strains, such as 129×1/SvJ, encode factors other than Nramp1 that contribute significantly to controlling bacterial replication within systemic tissues.

## Materials and Methods

### Ethics Statement

This study was carried out in strict accordance with the recommendations in the Guide for the Care and Use of Laboratory Animals of the National Institutes of Health. All protocols were approved by the Institutional Animal Care and Use Committee of the University of Washington.

### Generation of C57BL/6 Nramp-Cg mice

C57BL/6 mice consomic for A/J chromosome 1 were purchased from The Jackson Laboratory. These were then backcrossed to C57BL/6 mice and the presence of *Nramp1^G169^* was determined by sequencing. Marker-assisted breeding, also known as speed congenics, allowed us to monitor progressive replacement of flanking A/J sequences with C57BL/6 over the course of 5 backcross generations using in-house Illumina SNP genotyping [13]. Based on the SNP locations, crossover events occurred between bases 52,159,056 and 69,117,243 at the 5′ end, and between bases 76,984,491 and 85,664,769 at the 3′ end (from build 34 of the mouse genome). Thus Nramp-Cg mice encode between 7.9 and 33.6 Mbp of flanking A/J sequences in chromosome 1 (see Figure S1). These were then bred to homozygosity.

### Other mice

Nramp-Tg mice, generated by Philipe Gros [11] and provided to us by Ferric Fang, carry an Nramp1 transgene derived from 129sv genomic DNA [11]. Two single nucleotide polymorphisms have been identified within the Nramp1 gene between 129sv and A/J mice but neither SNP alters the amino acid sequence [14]. C57BL/6 and 129×1/SvJ mice were purchased from The Jackson Laboratory. Mice were bred and/or housed in a barrier facility at the University of Washington in ventilated racks with constant access to food and water.

### Real-time PCR

Bone-marrow derived macrophages were generated as previously described [15] and harvested after 7 days of culture. Total RNA was extracted using Trizol Reagent, (Invitrogen, Carlsbad, CA) followed by DNase treatment and cDNA synthesis. Oligo dT primed cDNA was reverse transcribed using Superscript II Reverse Transcriptase (Invitrogen, Carlsbad, CA), and Minus-RT controls were run for each sample. Relative Nramp1 expression levels were determined using Real-time PCR with Brilliant II SYBR Green qPCR reagent. The amplification was performed with an initial 2 min incubation at 50°C and 10 min at 95°C, followed by 40 cycles of 95°C, 15 s and 60°C, 1 min. Specificity of amplification was assessed using a melt-curve analysis and β-actin was used to normalize the data between samples. The Nramp1 primers were designed using Probe Finder v2.48 (Roche, Indianapolis, IN) yielding a 78 bp product with forward primer 5′-TAC CAG CAA ACC AAT GAG GA −3′ and reverse primer 5′- CCT GGG GAA GAT CTT AGC ATA GT-3′. β-actin primers were previously described [15].

### Bacterial infections

For IP infections, *S.* Typhimurium strain SL1344 was grown aerobically overnight in LB, pelleted, resuspended in PBS, and diluted to an appropriate concentration based on the O.D. at 600 nm. The inoculum was administered in 200 µl PBS intraperitoneally; actual CFU administered was verified by plating on LB plates (1600, 1770 and 1130 CFU respectively). For oral infections, *S.* typhimurium strain SL1344::lux (rederived in SL1344 from strain Xen 26; Caliper Life Sciences, now Perkin Elmer) was grown statically overnight in LB. Bacteria were enumerated using a Multisizer 4 (Beckman Coulter). Mice received either 5×10^7^ CFU (colonization experiments) or 1×10^8^ CFU (survival assays) in 200 µl in PBS +5% bicarbonate via oral gavage. Spleens, livers, and/or MLN were harvested at designated points post infection, homogenized, and serial dilutions were plated to determine the number of CFU per organ. Groups of 8–10 mice were infected orally with SL1344::lux for survival assays. Mice were weighed daily and scored for signs of clinical illness, i.e. ruffled fur, hunched posture and lethargy (scale of 0–3). At the start of the experiment, body weights were statistically indistinguishable between mouse strains (Figure S2A). Endpoint criteria for euthanasia were set at 20% weight loss (Figure S2B) or a score of 2.5 or greater in 2 out of 3 clinical categories.

### Statistics

Prism software (GraphPad, La Jolla, CA) was used for all statistical analyses with test indicated in the figure legend.

## Results

### Analysis of *Nramp1* expression in congenic and transgenic mice

**Figure 1:**
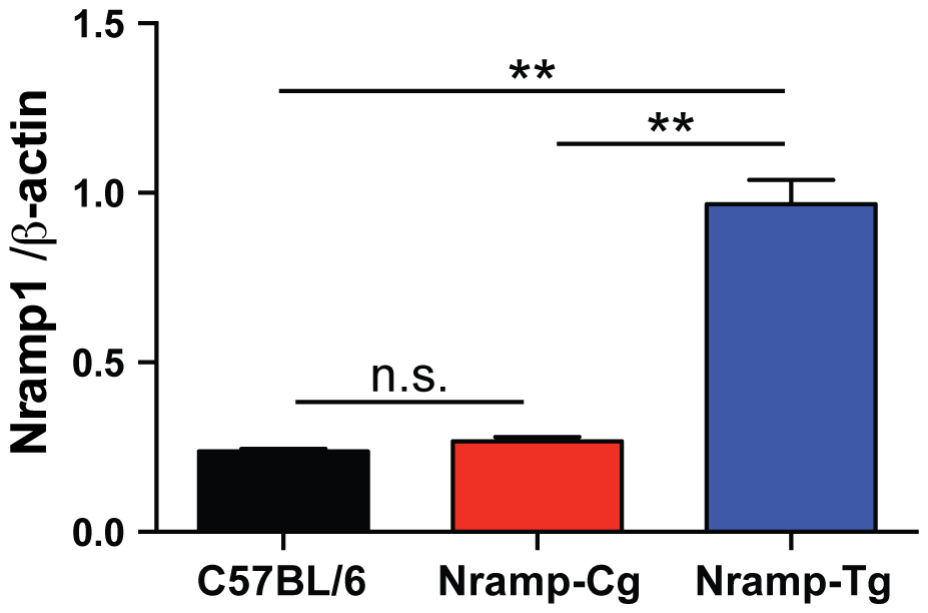
Relative expression of *Nramp1* in macrophages from C57BL/6, Nramp-Cg, and Nramp-Tg mice. Real-time PCR was performed on total RNA extracted from BMDMs cultured from indicated mouse strains and normalized to β-actin levels. Shown are the means of the results from 3 separate BMDM preparations. ** *P*<0.01, n.s. = not significant.

The *Nramp1* gene is actively transcribed in susceptible C57BL/6 mice [16]. However, the G169D missense mutation results in improper maturation/membrane integration and thus rapid degradation of the protein [3]. C57BL/6 mice that overexpress the resistant *Nramp1^G169^* allele from a transgene (Nramp-Tg) have been shown to survive acute intravenous infection with *S.* Typhimurium [11]. To address the question of whether overexpression of Nramp1 is required to control *Salmonella* infection, we generated a congenic C57BL/6 strain carrying the Nramp1 locus from chromosome 1 of A/J mice (Nramp-Cg). Relative RNA transcript levels were compared in bone marrow-derived macrophages cultured from C57BL/6, Nramp-Cg and Nramp-Tg mice. Macrophages were chosen for source RNA as they express high levels of Nramp1. We found that Nramp-Cg macrophages expressed equivalent levels of *Nramp1* RNA as C57BL/6 macrophages whereas Nramp-Tg macrophages expressed 4-fold more *Nramp1* RNA (Figure 1).

### Nramp1 is sufficient to control acute *S.* Typhimurium infection

**Figure 2:**
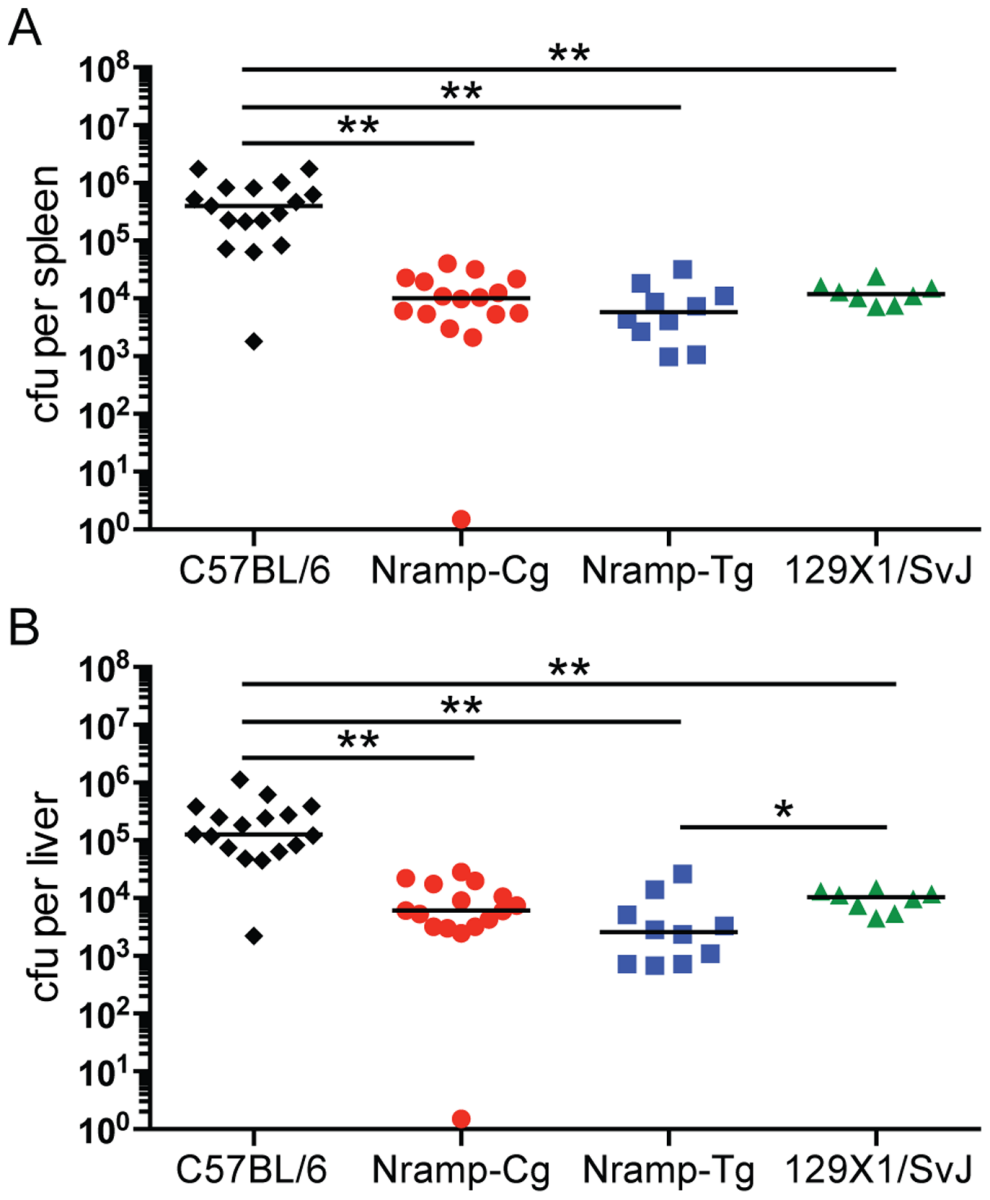
Nramp1+ C57BL/6 mice control systemic colonization during acute IP *Salmonella* infection. C57BL/6 (black diamond), Nramp-Cg (red circle), Nramp-Tg (blue square), and 129×1/SvJ (green triangle) mice were infected intraperitoneally and bacterial titers in the (A) spleen and (B) liver were measured 3 days later. Shown are the combined results from 3 independent experiments (lines indicate median values). Data were analyzed using the Mann-Whitney nonparametric t test. ** *P*<0.01, *P<0.05.

To determine whether congenic expression of *Nramp1^G169^* confers resistance to acute *Salmonella* infection, C57BL/6, Nramp-Cg, Nramp-Tg and 129×1/SvJ mice were challenged IP with virulent *Salmonella* strain SL1344 and bacterial colonization was measured in relevant organs (spleen and liver) 3 days later. As expected, bacterial titers in spleens and livers of 129×1/SvJ mice were significantly lower (40 fold) than in susceptible C57BL/6 mice (p<0.01) (Figure 2). Nramp-Cg and Nramp-Tg spleens were colonized at levels equivalent to that seen in 129×1/SvJ mice, with no observed *Nramp1* gene dosage effect. Liver colonization was lower in Nramp-Tg mice, reaching statistical significance relative to 129×1/SvJ but not Nramp-Cg (Figure 2B).

**Figure 3:**
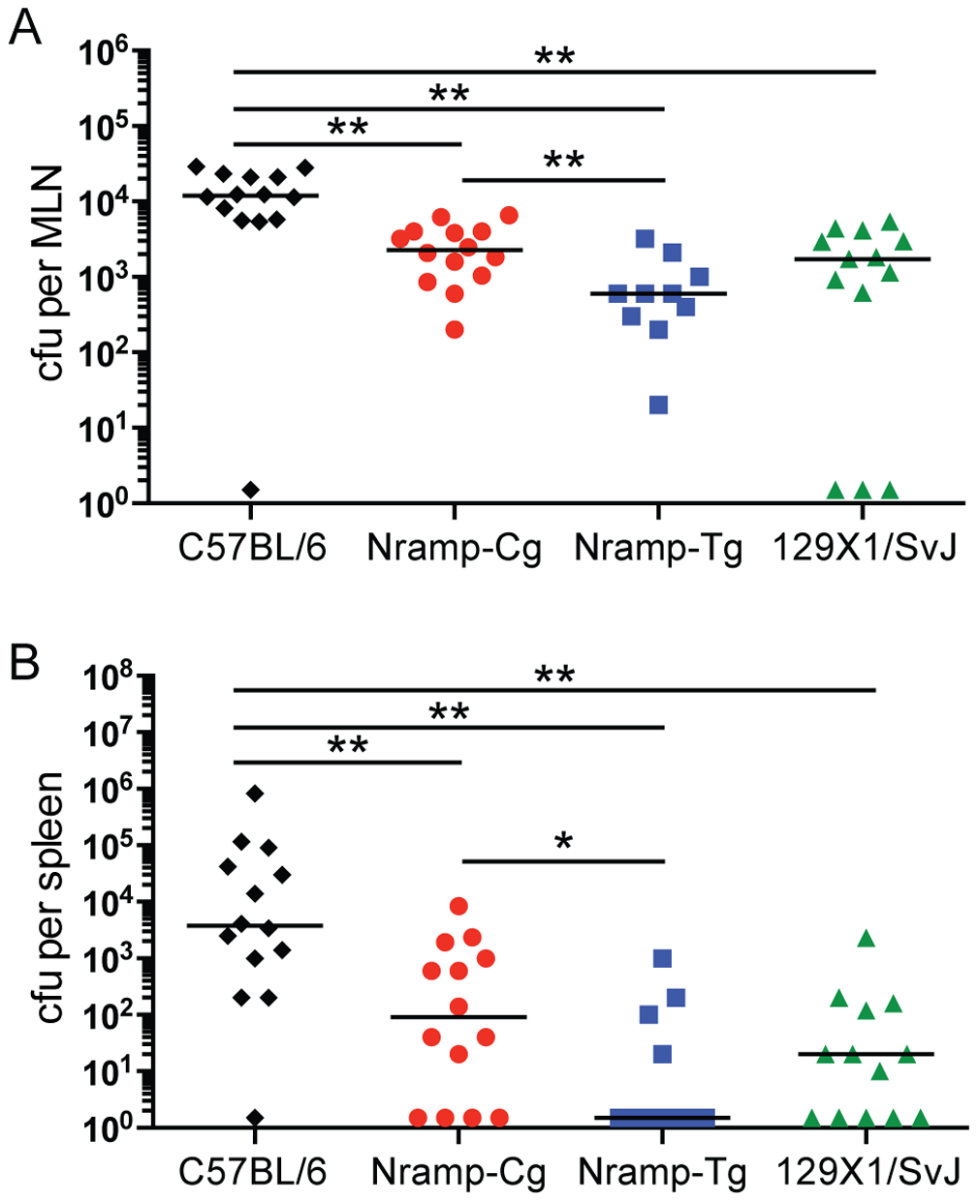
Dose dependent contribution of Nramp1 to control of acute oral *Salmonella* infection. C57BL/6 (black diamond), Nramp-Cg (red circle), Nramp-Tg (blue square), and 129×1/SvJ (green triangle) mice were infected orally and bacterial titers in the (A) MLN and (B) spleen were measured 5 days later. Shown are the combined results from 3 independent experiments (lines indicate median value). Data were analyzed using the Mann-Whitney nonparametric t test. ** *P*<0.01, *P<0.05.

While the IP infection model has been used extensively to study acute *Salmonella* infection, oral infection more closely mimics human disease. We therefore compared *Salmonella* colonization in C57BL/6, Nramp-Cg, Nramp-Tg and 129×1/SvJ mice during an acute oral infection. By day 5 post infection, expression of functional *Nramp1^G169^* in Nramp-Cg and Nramp-Tg mice led to better control of bacterial replication in both the MLN (5–10 fold lower) and spleen (>50 fold lower) compared to C57BL/6 mice expressing *Nramp1^D169^* (Figure 3), and was not significantly different from 129×1/SvJ mice. Significantly fewer bacteria crossed the intestinal mucosa and colonized the MLN of Nramp-Tg compared to Nramp-Cg mice (Figure 3A). In contrast to IP infection, spleens of orally infected Nramp-Tg mice contained 60-fold fewer *Salmonella* than spleens of Nramp-Cg mice (Figure 3B) indicating a dose-dependent contribution of Nramp1 expression to controlling systemic bacterial replication during the early stages of infection using a relevant GI model.

### While *Nramp1* expression enhances survival of C57BL/6 mice, additional genetic factors are required for long-term survival and systemic control of bacterial replication during persistent *Salmonella* infection

**Figure 4:**
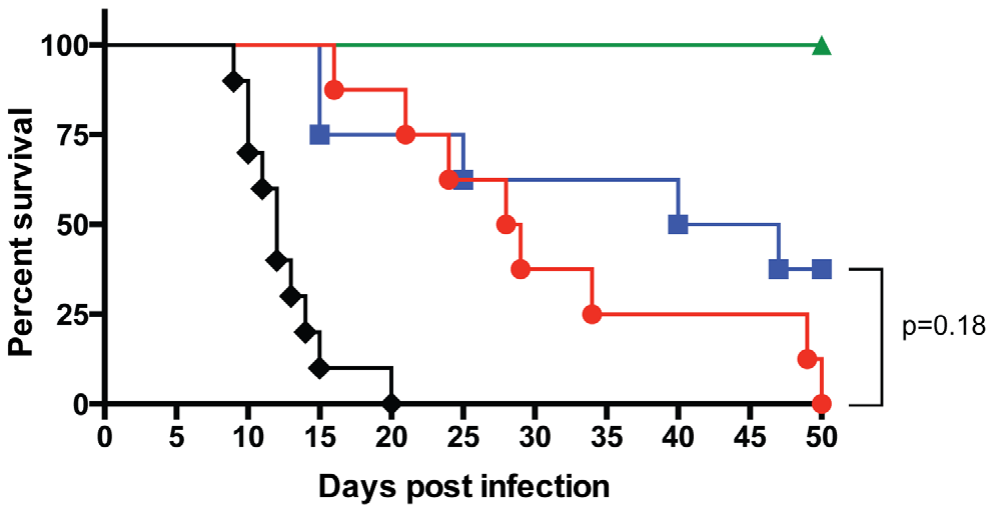
Nramp1 expression promotes survival of C57BL/6 mice, but Nramp1+ C57BL/6 mice eventually succumb to persistent *Salmonella* infection. C57BL/6 (black diamond; n = 10), Nramp-Cg (red circle; n = 8), Nramp-Tg (blue square; n = 8), and 129×1/SvJ (green triangle; n = 9) mice were infected orally with *Salmonella*. Mice were monitored daily, as described in Methods, and euthanized when they reached the designated endpoint. Shown are representative results from 2 independent experiments. Data were analyzed using the log rank (Mantel-Cox) test. All comparisons were highly significant (*P*<0.01) unless otherwise noted.

Since Nramp-Cg and Nramp-Tg mice control bacterial replication as well as 129×1/SvJ mice during the first 5 days, we asked whether C57BL/6 mice expressing *Nramp1^G169^* survive as well as 129×1/SvJ mice during the establishment of chronic *Salmonella* infection (Figure 4). As expected, susceptible C57BL/6 mice succumbed to oral infection with a median survival rate of 12 days. Median survival was significantly increased in Nramp-Cg, Nramp-Tg, and 129×1/SvJ mice (p<0.0001 for each compared to C57BL/6) (Figure 4). Interestingly, Nramp-Cg and Nramp-Tg mice succumbed to oral *Salmonella* infection more rapidly than 129×1/SvJ mice, which showed 100% survival at day 50 post infection, with median survival values of 29 and 44 days, respectively (p<0.01 for both Cg and Tg strains compared to 129×1/SvJ). These data show that Nramp1 expression is not sufficient to promote long-term survival of susceptible C57BL/6 mice.

### Tissue-specific contributions of Nramp1 to control of *S.* Typhimurium replication

**Figure 5:**
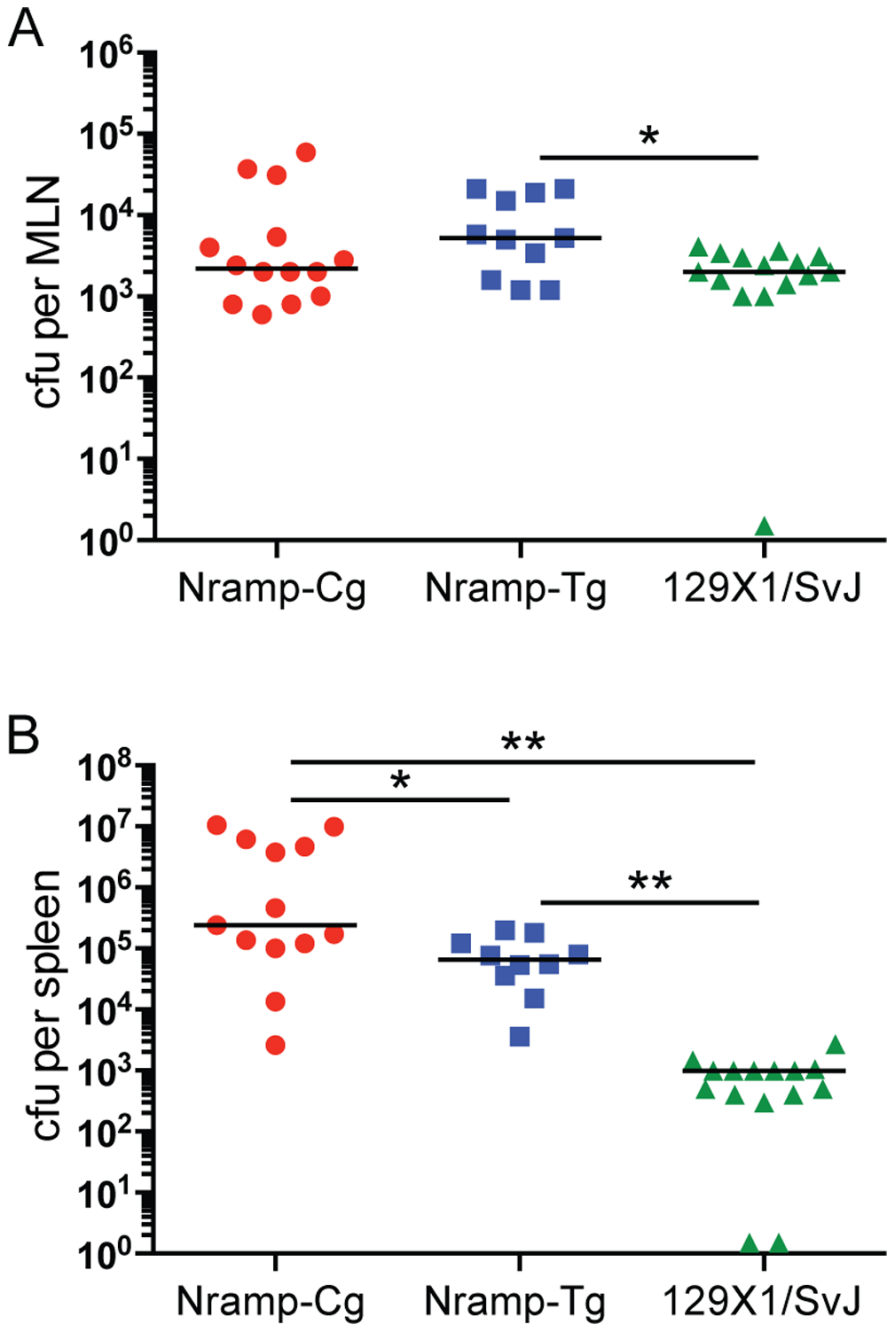
Tissue-specific contributions of Nramp1 expression for controlling bacterial replication. Nramp-Cg (red circle), Nramp-Tg (blue square), and 129×1/SvJ (green triangle) mice were infected orally and bacterial titers in the (A) MLN and (B) spleen were measured 30 days later. Shown are the combined results from 3 independent experiments (lines indicate median value). Data were analyzed using the Mann-Whitney nonparametric t test. ** *P*<0.01, *P<0.05.

The difference in survival between Nramp-Cg and Nramp-Tg mice did not reach statistical significance on day 50 (Figure 4), suggesting that Nramp1 overexpression in Nramp-Tg mice does not enhance the overall control of bacterial replication compared with Nramp-Cg hosts. However, median time to death does not provide information regarding anatomical restriction or tissue-specific control of bacterial replication. Therefore, we compared MLN and spleen colonization in Nramp-Cg, Nramp-Tg and 129×1/SvJ mice on day 30 post infection (Figure 5). Day 30 was chosen as a late time point where sufficient mice of each strain survived for tissue harvesting. Despite significant differences in survival on day 30 (Figure 4), we observed surprisingly equivalent colonization of the MLN across all Nramp1^+^ strains (Figure 5A) suggesting that expression of Nramp1 by resident macrophages is sufficient to control *Salmonella* replication in infected MLN. In contrast, splenic bacterial colonization levels differed significantly in each of the mouse strains tested (Figure 5B). Increased host Nramp1 expression (Tg>Cg) resulted in decreased splenic bacterial colonization (Tg<Cg). However, the spleens of both Nramp1+ C57BL/6 strains (Tg and Cg) harbored more *Salmonella* than spleens from 129×1/SvJ mice. These data suggest that host responses and/or selective pressures differ significantly between MLN and spleens. As a result, the contribution of Nramp1 expression to the restriction of systemic bacterial growth is anatomically distributed with host genes other than Nramp1 contributing to the ability of NTS-resistant 129×1/SvJ mice to effectively control splenic colonization.

## Discussion

Control of *Salmonella* replication *in vivo* is complex and differences in mouse genetic background can profoundly affect susceptibility to NTS (reviewed in [17]). Naturally-occurring mutations and targeted deletion of Nramp1 result in susceptibility to acute IP or IV infection [6], while expression of Nramp1 from a transgene promotes acute survival of otherwise susceptible mice [11]. We confirmed previous findings that Nramp1 expression is important for innate resistance during the acute phase of infection using the transgenic mice (Nramp-Tg) and congenic C57BL/6 mice carrying the Nramp1 locus from resistant A/J mice (Nramp-Cg) (Figure 2). While Nramp-Tg macrophages express approximately 4-fold more Nramp1 transcript than Nramp-Cg cells (Figure 1), we observed no Nramp1 dose-dependent difference in colonization between Nramp-Cg and Nramp-Tg mice during acute IP-induced *Salmonella* infection (Figure 2). However, when *Salmonella* was delivered orally, acute colonization was reduced in both the MLN and spleen of Nramp-Tg mice compared to Nramp-Cg mice (Figures 3). These data suggest that Nramp1 plays a more important role in controlling acute bacterial replication when the bacteria are transiting through the gut epithelium to reach systemic sites.

Despite early control of splenic colonization, Nramp1+ C57BL/6 mice did not survive chronic oral infection (Figure 4). While the resistant *Nramp1^G169^* allele is not sufficient to confer long-term survival to C57BL/6 mice, it does contribute to restriction of bacterial replication in MLN but not systemic sites, such as the spleen, suggesting that the function of macrophage-encoded *Nramp1^G169^* is anatomically compartmentalized. MLN are a critical tissue for restricting growth and dissemination of *Salmonella*. During trafficking from the gastrointestinal tract to the bloodstream, bacteria pass through the MLN, being brought there by gut-resident dendritic cells [8]. Within the MLN *Salmonella* are taken up by macrophages and most are degraded in an Nramp1-dependent manner [4], [10], [18]. However, some bacteria persist and replicate within MLN macrophages, thus creating a chronic reservoir of *Salmonella* responsible for relapsing infections [4], [10]. Mesenteric lymphadenectomised mice display increased spleen and liver colonization, increased severity of relapsing infection and increased mortality following oral inoculation, thus demonstrating the importance of MLN as filters protecting systemic tissues [8], [10]. Equivalent CFU in chronically infected Nramp-Cg, Nramp-Tg and 129×1/SvJ MLN (Figure 5A) suggests that expression of Nramp1 in MLN macrophages is sufficient to control *Salmonella* replication in this tissue. Why then is Nramp1 expression not sufficient to control splenic colonization?

As in MLN, macrophages are the major cell type supporting *Salmonella* replication in the spleen [4], [19]. However, within chronically infected spleens *Salmonella* preferentially survive and replicate within a subset of macrophages, called hemophagocytic macrophages, that have ingested non-apoptotic cells of hematopoietic lineage, but are killed by macrophages that have phagocytosed nothing or have phagocytosed dead host cells [19], [20]. Nramp1 expression in hemophagocytic macrophages was not tested, but the fact that *Salmonella* are able to replicate efficiently in these cells suggests that Nramp1, if expressed at all, is not playing a crucial role in controlling bacterial replication within splenic macrophages. Increased Nramp1 copy number did reduce splenic CFU in Nramp-Tg mice compared to Nramp-Cg mice, but bacterial titers were still 66-fold greater than in 129×1/SvJ mice (Figure 5B), demonstrating an important new finding of our study that genes other than Nramp1 are required for systemic control of chronic *Salmonella* infection. Our data demonstrating that uncontrolled splenic colonization negatively impacts host survival is consistent with studies examining asplenic humans. Patients with functional or anatomic asplenia are at significantly increased risk of overwhelming infection with mortality rates of 50–70% [21]. Interestingly, *Salmonella* species are the leading cause of infection in patients with sickle cell disease, an important cause of functional asplenia [22].

Studies examining phenotypic and genetic differences between susceptible and resistant mouse strains have identified a large number of loci (other than Nramp1) involved in both innate and adaptive immune mechanisms [23]–[30]. For example, comparisons between the response of Nramp-Tg and Sv129S6 mice to chronic *Salmonella* infection showed that Nramp-Tg mice experience more severe inflammatory disease than Sv129S6 mice, with higher levels of proinflammatory serum cytokines (IFNγ, TNFα, IL-1β and IL-2) and chemokines (MCP-1 and CXCL1) and decreased anti-inflammatory cytokines (IL-10 and IL-4)[27]. While enhanced Th1 responses should promote bacterial clearance in Nramp-Tg spleens, uncontrolled inflammation likely contributes to the increased mortality seen in Nramp1+ C57BL/6 mice (Figure 4).

Adaptive immunity is required for resistance late in infection [31], [32]. Control of bacterial replication in the spleens of 129SvJ × C57BL/6 F1 mice correlates with robust T cell effector function and reduced immune suppression by regulatory T cells [33]. While both C57BL/6 and 129×1/SvJ mice share the same MHC haplotype, differential regulation of T cell responses may account for the ability of 129×1/SvJ mice to control *Salmonella* replication better/longer than Nramp1+ C57BL/6 mice. Natural killer (NK) cells have recently been shown to regulate T cell immunity via a number of different strategies, including cytokine secretion and perforin-mediated T cell death (reviewed in [34]). Activation of NK cells is controlled by the Ly49 family of class I binding receptors. Of relevance to this study, genetic analysis of the Ly49 gene cluster in 129/J mice identified extensive differences in gene content relative to C57BL/6 mice [29] that correlated with altered NK cell activation in 129/J mice [30]. Additional studies comparing genetic susceptibility to chronic *Salmonella* infection using the Nramp1+ mouse strains described here would enhance our understanding of factors required for control of acute *Salmonella* infection and the establishment of persistent infection.

## Supporting Information

Figure S1 **Location of SNP identifying recombination junctions in Nramp-Cg mice.** Illumina SNPs that are either of C57BL/6 (black) or A/J (red) origin are shown on a section of Chromosome 1 (outer SNP nucleotide numbers according to Build 34 are indicated in parentheses). Distance between SNP indicated in Mb.
(TIF)

Figure S2 **Comparison of body weight over the course of infection.** Average body weight of mice used in Figure 4 (survival). A) Starting weight (in grams) on day 0. Data were analyzed by one-way ANOVA and differences found to be insignificant. B) Weight change over the course of the experiment. At each timepoint, the weight of each mouse was compared to its weight on day 0 and the difference recorded as percent weight change. Shown are average changes in weight over 50 days of infection. During analysis, mice that succumbed to infection were represented by their last recorded weight for the remainder of the timecourse. C57BL/6 (black diamond; n = 10), Nramp-Cg (red circle; n = 8), Nramp-Tg (blue square; n = 8), and 129×1/SvJ (green triangle; n = 9).
(TIF)
