# Correction: Temporal and Anatomical Host Resistance to Chronic *Salmonella* Infection Is Quantitatively Dictated by Nramp1 and Influenced by Host Genetic Background

**DOI:** 10.1371/journal.pone.0117648

**Published:** 2015-01-21

**Authors:** The PLOS ONE Staff

The name of the mouse cell line 129x1/SvJ appears incorrectly throughout the manuscript. The correct name should be 129X1/SvJ.

## References

[1] Loomis WP, Johnson ML, Brasfield A, Blanc M-P, Yi J, et al (2014) Temporal and Anatomical Host Resistance to Chronic *Salmonella* Infection Is Quantitatively Dictated by Nramp1 and Influenced by Host Genetic Background. PLoS ONE 9(10): e111763 doi: 10.1371/journal.pone.0111763 25350459 10.1371/journal.pone.0111763 PMC4211889

