# Supplementary figures and images for: Temporal and Anatomical Host Resistance to Chronic Salmonella Infection Is Quantitatively Dictated by Nramp1 and Influenced by Host Genetic Background
Source: PLoS One. 2014 Oct 28;9(10):e111763. doi: 10.1371/journal.pone.0111763 (PMC4211889; doi:10.1371/journal.pone.0111763)

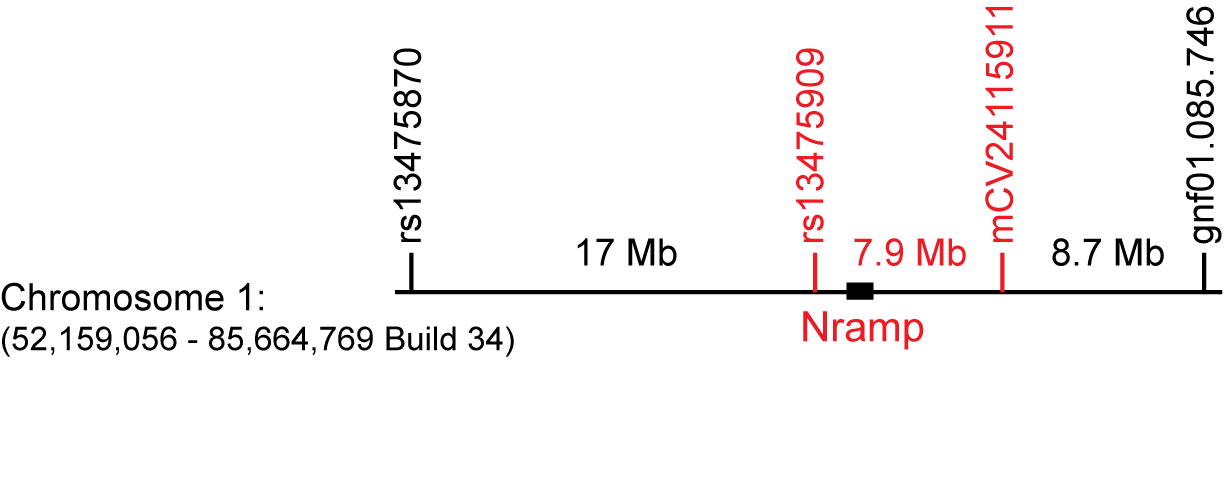

Supplement: Figure S1 — Location of SNP identifying recombination junctions in Nramp-Cg mice. Illumina SNPs that are either of C57BL/6 (black) or A/J (red) origin are shown on a section of Chromosome 1 (outer SNP nucleotide numbers according to Build 34 are indicated in parentheses). Distance between SNP indicated in Mb. (TIF) [file pone.0111763.s001.tif]

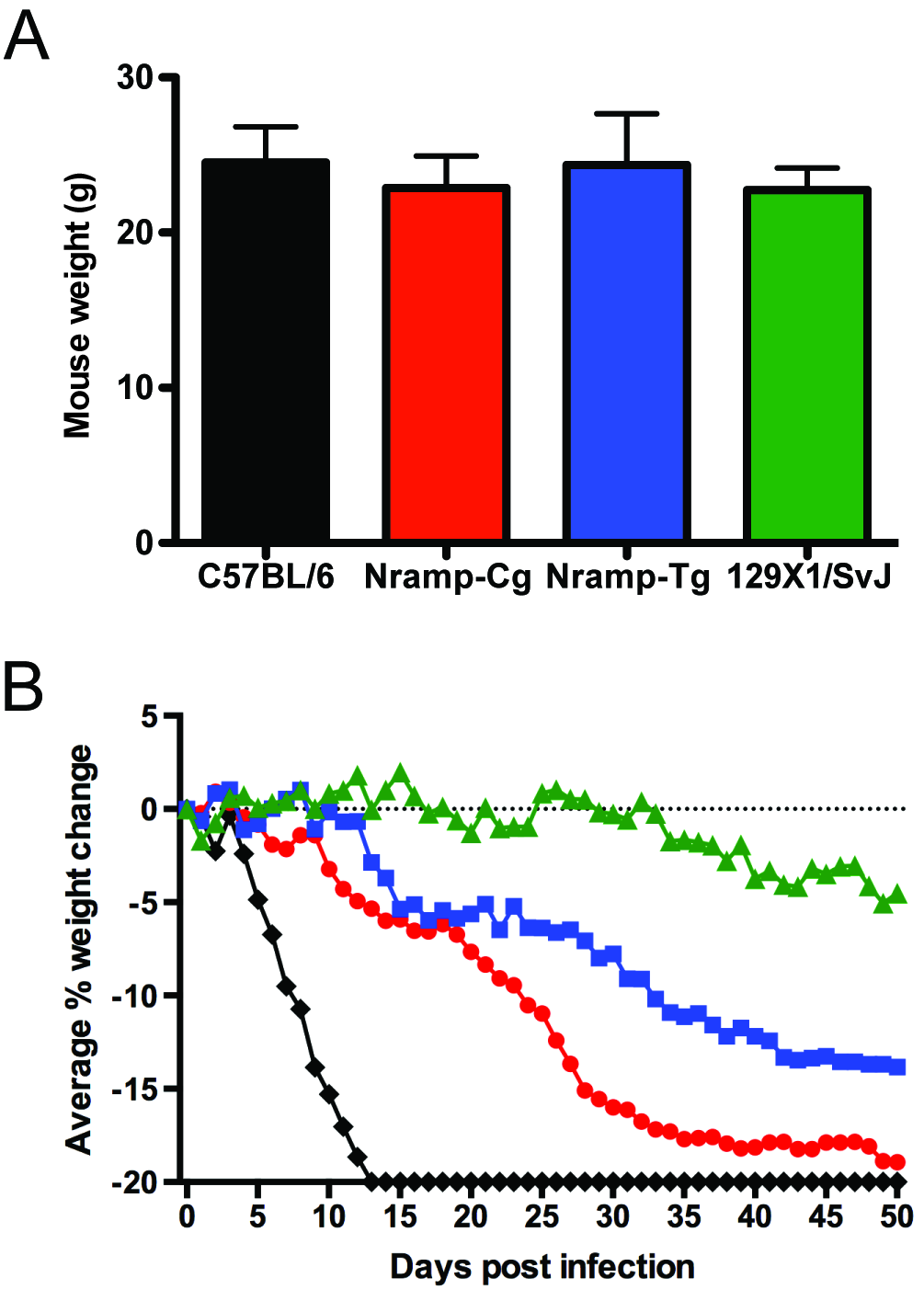

Supplement: Figure S2 — Comparison of body weight over the course of infection. Average body weight of mice used in Figure 4 (survival). A) Starting weight (in grams) on day 0. Data were analyzed by one-way ANOVA and differences found to be insignificant. B) Weight change over the course of the experiment. At each timepoint, the weight of each mouse was compared to its weight on day 0 and the difference recorded as percent weight change. Shown are average changes in weight over 50 days of infection. During analysis, mice that succumbed to infection were represented by their last recorded weight for the remainder of the timecourse. C57BL/6 (black diamond; n = 10), Nramp-Cg (red circle; n = 8), Nramp-Tg (blue square; n = 8), and 129×1/SvJ (green triangle; n = 9). (TIF) [file pone.0111763.s002.tif]
